# Exploratory Co-Design on Electronic Health Record Nursing Summaries: Case Study

**DOI:** 10.2196/68906

**Published:** 2025-03-11

**Authors:** Suhyun Park, Jenna L Marquard, Robin R Austin, Christie L Martin, David S Pieczkiewicz, Connie W Delaney

**Affiliations:** 1 Cizik School of Nursing, The University of Texas Health Science Center at Houston, #557, 6901 Bertner Ave, Houston, TX, 77030, United States, 1 713-500-2246; 2 School of Nursing, University of Minnesota, Minneapolis, MN, United States; 3 Institute for Health Informatics, University of Minnesota, Minneapolis, MN, United States

**Keywords:** electronic health records, interview, nurses, user-computer interface, co-design

## Abstract

**Background:**

Although electronic health record nursing summaries aim to provide a concise overview of patient data, they often fall short of meeting nurses’ information needs, leading to underutilization. This gap arises from a lack of involvement of nurses in the design of health information technologies.

**Objective:**

The purpose of this exploratory co-design case study was to solicit insights from nurses regarding nursing summary design considerations, including key information types and the preferred design prototype.

**Methods:**

We recruited clinical nurses (N=33) from 7 inpatient units at a university hospital in the Midwestern United States using a purposive sampling method. We used images from a simulated nursing summary to generate visual card versions of the 46 information types currently included in an electronic health record vendor–generated nursing summary. Participants selected which cards to include and arranged them in their designs based on their perceived relevance of the information types to the summary and their preferred reading layout. The nurses’ perceived relevance of information types to the summary was analyzed by quantifying the frequency of included cards, while the nurses’ preferred reading layout was analyzed by quantifying the occurrence of closely paired cards to identify common groupings. After participants evaluated the information type cards, debriefing interviews were conducted and analyzed thematically to explore their rationales for the desired content and its arrangement.

**Results:**

The participants demonstrated a high level of engagement in the activities. On average, all 33 participants included 61% (n=28) of the total information types (n=46). The most frequently included cards were “unit specimen” (results of the analysis of body fluid, tissue, or urine), “activity,” “diet,” and “hospital problems,” each included by 33 participants. Participants most frequently preferred adjacency of the following pairs: “activity” and “diet” (paired by 26 participants; 79%) and “notes to physicians” and “notes to treatment team” (paired by 25 participants; 76%). Participants preferred arranging the cards to improve information accessibility, focusing on key information types.

**Conclusions:**

Involving nurses in the co-design process may result in more useful and usable designs, thereby reducing the time required to navigate nursing summaries. Future work should include refining and evaluating prototypes based on the designs created by the nurses.

## Introduction

### Background

Ineffective communication among clinicians is a leading cause of sentinel events, such as falls and delays in treatments [1]. Given time constraints, nurses review vast amounts of patient information in electronic health records (EHRs) at the beginning of each shift; thus, effective tools for summarizing patient information are crucial [2]. Nursing summaries in EHRs are intended to provide a concise overview of essential patient data (eg, vital signs, intake and output, weight) on a single page, but rigid and incomplete formats have led to underutilization [3], resulting in nurses developing highly variable methods, such as paper notes, for summarizing patient information.

End-users are involved in the EHR development phase to prevent low usability and support optimal EHR utilization [4]. Co-design (or participatory design) has gained attention as a strategy to integrate stakeholder viewpoints, bridging the gap between tools and users’ needs to ensure the long-term adoption of tools [5]. Co-design aims to actively involve potential user groups in designing tools and interventions, leveraging their lived experiences to tailor outputs to their expressed needs and preferences, while aligning with existing workflows and available resources [6]. In health care, co-design has been applied across various settings and user groups, including in the creation of predictive analytics dashboards and decision support systems to enhance care quality and client outcomes in aged care [6]. Co-design has also been used to examine clinical nurses’ general ideas about information display design in a nursing summary [2] and to develop a mobile health application to assist patients with obesity in self-managing their preparation for elective surgery [7].

The use of information cards as a tool for stakeholder engagement is an approach adopted by designers to structure the co-design ideation process [8,9]. Card sorting, a prevalent user-centered design technique, actively involves participants categorizing labeled cards based on criteria that most resonate with them [10]. Card sorting offers numerous benefits, including facilitating the organization of complex information into intuitive groupings, promoting consensus building among diverse user groups, enhancing the user experience by ensuring content and functionality are logically structured, and providing valuable insights into users’ preferences and priorities [11]. Although we did not fully follow the card sorting technique, we adapted key principles from the card sorting method, such as labeling and analyzing the arrangement of cards, to suit this study context. This exploratory research focused on nurses’ perceived usability and their feedback to inform nursing summary design strategies. The design strategies informed by nurse users’ input could streamline navigational pathways, thereby reducing cognitive load. This aligns with the goals of the National Burden Reduction Collaborative, which unites health care organizations, federal agencies, and standards organizations to reduce clinical burden nationwide [12].

### Problem Statement

Nurses’ participation in EHR design is often limited, despite their role as key users [2,13]. A deeper understanding of their unique requirements and suggestions for EHR design is needed. Recognizing the pivotal role of end-user involvement in design [5], we used a co-design activity to examine nurses’ perspectives on the use and arrangement of information types (eg, vital signs, intake and output, weight) within an existing EHR vendor’s nursing summary. Specifically, the co-design activity aimed to gain insights into nurses’ perspectives on nursing summary design, including which information types to include and how to arrange the information types.

## Methods

### Study Design

This was an exploratory, single session of co-design activities that gathered nurses’ feedback and identified their design preferences. Although conducted independently, this study was part of an eye-tracking simulation, which focused on examining nurses’ visual attention to EHR nursing summary information types (submitted to another journal).

### Participants and Recruitment

We recruited participants from 5 adult medical-surgical and 2 intermediate care units at a university hospital using a purposive sampling method. We aimed to recruit 30 or more participants, as this is recommended for eye-tracking simulations. These units used the same summary layout as the example summary employed in this study. To be included, participants must have had at least 12 months of experience as a registered nurse. We excluded nurses who were not in frontline patient care, such as nurse educators, nurse managers, and nurse research coordinators. Recruitment was facilitated by nurse managers, who distributed email flyers providing details about the study.

### Data Collection

**Figure 1.**
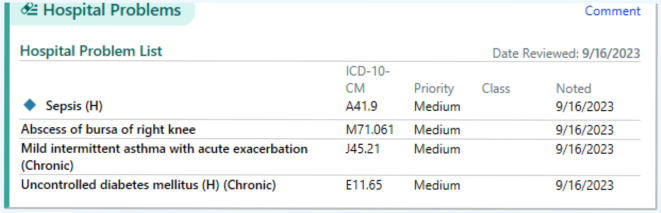
Example of an information type card. Each information type card used a consistent design, including a colored boundary, heading, and optional content.

Participants were individually invited to a shared research space from August to September 2023. We used a nursing summary from a simulated patient case on a training platform that mirrored the EHR system used by the recruiting hospital. Since the hospital had used the Epic EHR system (Epic Systems) since 2011, we assumed all participants were familiar with its layout. The EHR vendor–generated nursing summary included 46 information types (eg, vital signs, intake and output, weight). We printed and cut out cards for each information type so the participants could move them around as they liked (Figure 1). See Multimedia Appendix 1 for the full original nursing summary layout and Multimedia Appendix 2 for details of the information types. A lead researcher (SP) conducted all co-design activities independently, while a co-author (JLM) who is a human factors expert provided guidance on the study design and monitored the co-design activities after each session.

**Figure 2.**
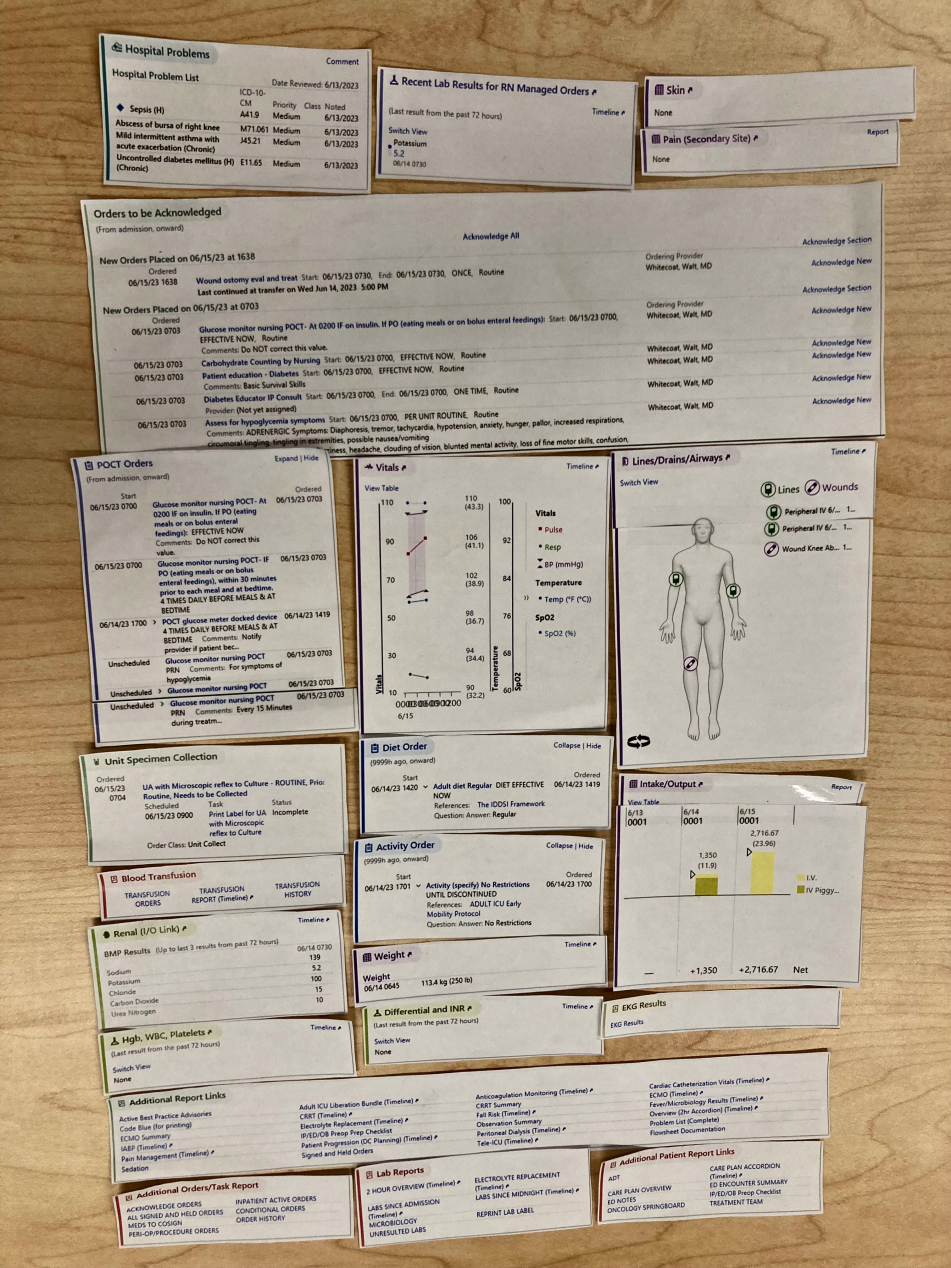
A sample summary design prototype by a participant. The layout shows the information types to include and their preferred arrangement.

First, each participant reviewed each information type card to determine whether it was important enough to be included in the summary. Participants then arranged the included cards they wanted to present in the nursing summary on a desk to develop their preferred layout for the nursing summary screen. We clarified that the focus of the study was on determining the relevance of specific types of information (ie, social determinants of health) in the context of a nursing summary and arranging them, rather than the graphical design or the content of the information within the cards. We photographed each participant’s design prototype for subsequent analysis. Figure 2 shows an example of a design prototype created by one participant. Following the activity, a debriefing interview was conducted to collect feedback, and each conversation was audio-recorded via Zoom for subsequent transcription and analysis. Participants were prompted with the following guided questions: (1) explain your reasoning process in relation to the design layout; (2) discuss any specific considerations you made regarding information arrangement; and (3) offer any additional comments on your design.

We collected demographic information through a survey administered after the debriefing interviews.

### Ethical Considerations

The study protocol was approved by the University of Minnesota Institutional Review Board (STUDY00018047). The principal investigator (SP) provided verbal and written information about the study to the participants and obtained verbal consent, as the requirement for written consent was waived. All data collected during the study were deidentified and securely stored in a university cloud-based storage system. The audio recordings and transcripts were deleted after the data analysis was completed. As this study was a part of an eye-tracking simulation, participants received a US $65 gift card for their time and effort upon completing all activities.

### Data Analysis

We evaluated the relevance of each information type to the summary by quantifying the number of nurses who chose to include the information type card in the design output. We then quantified the frequency of card pairs to identify which information type cards nurses tended to place next to each other in their design prototype, providing insights into the cohesive grouping of information [11]. One researcher (SP) reviewed the deidentified transcripts of the debriefing interviews for accuracy and coded for thematic analysis. The codes and overarching themes were reviewed in weekly meetings with another researcher (JLM).

## Results

### Sample Characteristics

The characteristics of the 33 participants are presented in Table 1. More than half (n=19, 58%) of the participants were 25‐44 years old, 85% (n=28) were female, 76% (n=25) were White, and none were Hispanic. Most nurses included in this analysis had at least a baccalaureate degree related to nursing (n=30, 91%). The participants generally had fewer years of nursing experience and worked fewer hours compared to a representative sample of national nurses (median=13 years and median=37 hours, respectively) [14].

**Table 1.** Baseline characteristics of the participants (N=33).

Characteristic	Value
Age (years), n (%)	
18‐24	9 (27)
25‐44	19 (58)
45‐64	5 (15)
≥65	0 (0)
Sex, n (%)	
Male	5 (15)
Female	28 (85)
Other	0 (0)
Race, n (%)	
Black or African American	5 (15)
White	25 (76)
Asian	2 (6)
Native American or Alaskan	0 (0)
Other	0 (0)
Prefer not to say	1 (3)
Ethnicity, n (%)	
Hispanic or Latinx	0 (0)
Non-Hispanic	33 (100)
Other	0 (0)
Relationship status, n (%)	
Single	14 (42)
Married	14 (42)
Partnered	2 (6)
Divorced	2 (6)
Separated	1 (3)
Highest nursing degree, n (%)	
Diploma	3 (9)
BSN	28 (85)
MS or MSN	2 (6)
PhD or DNP	0 (0)
Hours worked per week, median (IQR)	36 (30-36)
Years of registered nurse experience, median (IQR)	4 (1.5-8)
Years of using electronic health records, median (IQR)	4 (1.5-8)

### Card Frequency

**Figure 3.**
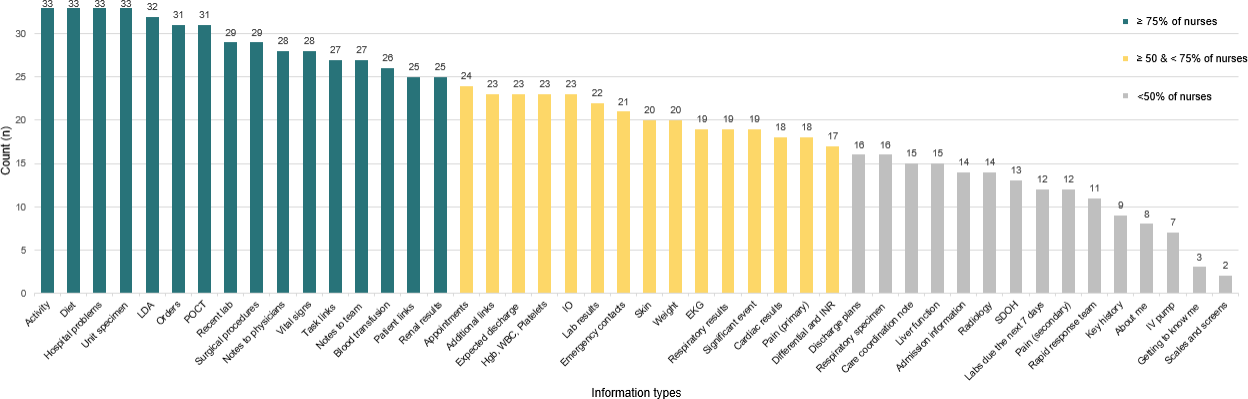
Frequency of included cards. Participants selected information type cards that they think are relevant to include in nursing summaries. EKG: electrocardiogram; Hgb: hemoglobin; INR: international normalized ratio; IO: intake and output; IV: intravenous; LDA: lines, drains, and airways; POCT: point-of-care testing; SDOH: social determinants of health; WBC: white blood cell.

On average, participants included 28 of the 46 information type cards in their design prototypes. Notably, all participants (N=33) included “unit specimen” (results of the analysis of body fluid, tissue, or urine), “hospital problems,” “diet,” and “activity” in their design prototype (Figure 3). However, certain information type cards were included less frequently: fewer than 50% of participants included 15 of the information type cards, and fewer than 10 participants included “key history and social determinants” (n=9), “about me” (individualized notes) (n=8), “intravenous pump settings” (n=7), “patient history notes” (n=3), and “scale and screen documentation” (n=2).

### Card Adjacency

**Figure 4.**
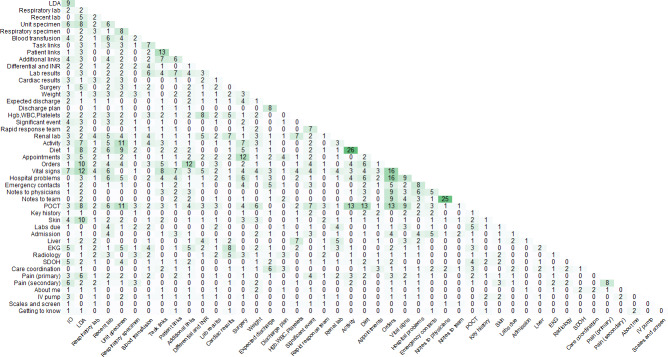
Item-by-item matrix of card adjacency. The frequency of adjacency was manually counted when 2 cards were placed next to each other with no third card in between. EKG: electrocardiogram; Hgb: hemoglobin; INR: international normalized ratio; IO: intake and output; IV: intravenous; LDA: lines, drains, and airways; POCT: point-of-care testing; SDOH: social determinants of health; WBC: white blood cell.

Figure 4 shows the number of participants who placed each pair of information type cards next to each other in their design. Darker shading was used to highlight cells where 2 information type cards were located next to each other more frequently. The pairs of information type cards located next to each other most frequently were “activity” and “diet” (n=26, 79%), followed by “notes to physicians” and “notes to treatment team” (n=25, 76%). Other pairs of cards that were placed next to each other included “orders” and “hospital problems” (n=16, 48%) and “orders” and “vital signs” (n=16, 48%). “Orders” and “hospital problems,” and “orders” and “vital signs” are not located next to each other in the current EHR design.

### Interview Findings

Table 2 presents themes, subthemes, and exemplary quotes derived from the debriefing interviews. The participants emphasized the importance of readability in their design choices, opting for a layout that facilitates top-to-bottom reading of key information based on priority. They noted that grouping related information together would facilitate information scanning. They also prioritized identifying immediate action items (eg, physicians’ orders) over delving into patient characteristics, such as social determinants of health. Owing to the typical time constraints needed to review patient information at the beginning of a shift, participants removed redundant information cards that hindered a swift and effective patient overview. Last, beyond the given design layout, participants provided design-related commentaries, pointing out that some information types required unnecessary clicks to get to the desired information, which limited usability.

**Table 2.** Common themes, subthemes, and supporting quotes from debriefing sessions based on their co-design activities, uncovering participants’ reasoning process and design considerations.

Themes and subthemes	Quotes
Improving information accessibility	
Key information positioned upward	“I have it from top to bottom. So it’s the least important thing towards the bottom.” [P11] “Diet is not as important, so I kept it going to the bottom.” [P24] “I usually place it at the top of the page, not necessarily because it’s important, but because it needs to be visible for everyone to see…Otherwise, no one else will notice it. Not everyone will scroll down.” [P4] “Most important would be at like the top, in a top-down order.” [P1] “I like that the orders are at the top because they’re the most important; they’re what the doctor wants us to be doing with the patient.” [P9]
Proximity of similar information	“I want lab results in the same spot.” [P24] “That’d be nice if they were together. Orders around orders. Orders can stick together.” [P1]
Focusing on key information types	
Go-to action items over background details	“I believe that social determinants of health are important. However, during my admission process, I do not check the social determinants of health. It is crucial for social workers and us to better understand the patient, but it is not my initial priority. When I first receive patients, I do not check the social determinants of health.” [P26] “I always want to know what orders are because these would be done right away.” [P22]
Removing redundancy	“The additional report things have a fall risk timeline, but then there’s a fall risk injury (information card). I just feel like it’s totally redundant.” [P22] “The card, ‘Getting to know me,’ I feel like this was redundant with ‘About me.’ I honestly never use this.” [P5]
Easy-to-use design	
Unnecessary steps to the desired information	“We cannot see the treatment team unless we do another click here and check the treating team.” [P26] “...a lot of people have a hard time with, like, everything’s here but you have to click a lot to get out of here, right? If there’s a transfusion order, this shouldn’t be bold or an asterisk (on the order name). There should be an order you need to give blood or something that you could click on…Why do you need extra things to press?” [P22] “I really don’t use this summary page that much, because it’s just so overwhelming. I guess it’s just too many things there that I don’t even know, like how to navigate it, which is why I made my system, which is more clicking.” [P5]

## Discussion

### Principal Results

The co-design case study highlights the importance of aligning EHR nursing summary design with nurses’ cognitive workflows, demonstrating that nurses prioritize and organize information in ways that make sense to them. Concise, relevant information allows nurses to quickly review a patient’s status, while strategically grouping related data helps them navigate and interpret complex information more efficiently. The current nursing summary contains overwhelming amounts of information that is often presented ineffectively, necessitating extensive searching to consolidate scattered data. The findings indicate a significant need to remove less relevant information from the summary. Also, nurses emphasized the importance of “unit specimen” (results of the analysis of body fluid, tissue, or urine), “activity,” “diet,” and “hospital problems” in their design prototype, suggesting these are the key information types for patient overviews. Nurses tended to prioritize obtaining information on current problems, daily living status, or treatment plans. Medical history or miscellaneous notes can be a source of information but are not always necessary for the initial patient overview. Our findings are consistent with prior work in which clinicians used a limited number of clinical information concepts at the time of patient admission, suggesting better electronic data management strategies, including the priority display of frequently used clinical concepts [15].

Participants strategically arranged information cards to align with their cognitive workflow by placing them adjacent to other relevant cards. The adjacency of information often signifies the similarity or relative importance of the information [16]. Previous studies have also emphasized the benefits of placing paired EHR information adjacent to each other on the screen to facilitate user interface navigation [17-19]. Some information pairs nurses identified with high frequency did not align with the current nursing summary layout. For example, “orders” and “hospital problems,” “orders” and “vital signs,” “orders” and “point-of-care-testing order” (tests that are conducted at the patient’s bedside), and “point-of-care-testing order” and “activity” were frequently located next to each other in the participant designs, but not in the existing layout. In addition to the adjacent placement of relevant information, top-to-bottom organization of information was commonly found in the designs. The placement of items at the top of the screen naturally calls attention to those items [20]. Instead of scrolling down for details on the screen, nurses tended to place good-to-know but not essential information toward the bottom of their designs. The concise and cognitively aligned design supports nurses in narrowing their focus from the comprehensive patient record to a targeted subset of relevant information, thereby enhancing the efficiency of initial assessments and ensuring that pertinent details are readily accessible when required [21].

### Strengths and Limitations

These findings show the value of co-design activities that involve nurses in the design process—to create an EHR nursing summary that better supports their needs. By prioritizing the most relevant patient information and streamlining the data presentation, the study offers design strategies that could alleviate the EHR burden of navigating from one piece of information to another. The identified design strategies could enhance efficiency and support the National Burden Reduction Collaborative’s mission to reduce the overall burdens on health care professionals [12].

While this case study successfully identified nurses’ information preferences aligning with their workflow, it has limitations. The use of a single simulated patient case for the co-design activities and the small sample size from a single institution may limit the generalizability of the findings. Due to the small sample size, we did not group participants by demographics (eg, unit-specific role, shift, work experience). While this study aimed to explore general information needs and design considerations for nursing summaries by including participants from multiple units, nurses’ specific needs may vary based on their characteristics. For example, nurses in surgical units may prioritize surgery-related information, while nurses in general medical units may focus on different aspects. Additionally, night shift nurses may prioritize information about upcoming examinations for the next day. Nurses’ characteristics may also influence their priorities and responsibilities, underscoring the importance of considering these variables in future research.

### Conclusion

This co-design case study provides valuable insights into nurses’ preferences and strategies for organizing information in an EHR nursing summary, underscoring the need to redesign the layout based on its practical use. We emphasized the significance of reducing less relevant information and optimizing the layout according to nurses’ information priorities. These efforts have the potential to improve the usefulness of the current nursing summary. The direct involvement of nurses as co-designers holds promise for developing user-centered EHR tools that support nursing cognition, decision-making, and workflow.

## Supplementary material

10.2196/68906 Multimedia Appendix 1 Nursing summary layout.

10.2196/68906 Multimedia Appendix 2 Descriptions of information types in nursing summaries.

## Abbreviations

EHR: electronic health record
