# Supplementary material for: Exploratory Co-Design on Electronic Health Record Nursing Summaries: Case Study
Source: JMIR Form Res. 2025 Mar 11;9:e68906. doi: 10.2196/68906 (PMC11918977; doi:10.2196/68906)
Supplement: Multimedia Appendix 1 [file formative-v9-e68906-s001.pdf]

Summary

Nursing Summary | Inpatient Active Orders | Event Log | Intake/Output

Nursing Summary | Off

Select Time Range

Kristoff, Kingsley #115000002 (CSN:180959) (25 year old M) (Adm: 09/17/23)

JNP1-TRN IPCD Med Surg-TRN IPCD Med Surg

Sticky Notes to Physicians

Comment

Treatment Team Sticky Notes

Comment

Click to Update Discharge Info

Expected Discharge Date

Expected Date/Time: 9/20/2023

Discharge Milestones

- Place Discharge Order
- Complete med reconciliation
- Print AVS

Expected Discharge History

Additional Report Links

Active Best Practice Advisories	Adult ICU Liberation Bundle (Timeline)	Anticoagulation Monitoring (Timeline)	Cardiac Catheterization Vitals (Timeline)
Code Blue (for printing)	CRRT (Timeline)	CRRT Summary	ECMO (Timeline)
ECMO Summary	Electrolyte Replacement (Timeline)	Fall Risk (Timeline)	Fever/Microbiology Results (Timeline)
IABP (Timeline)	IP/ED/OB Preop Prep Checklist	Observation Summary	Overview (2hr Accordion) (Timeline)
Pain Management (Timeline)	Patient Progression (DC Planning) (Timeline)	Peritoneal Dialysis (Timeline)	Problem List (Complete)
Sedation	Signed and Held Orders	Tele-ICU (Timeline)	Flowsheet Documentation
Wavestrips			

Orders to be Acknowledged

(From admission, onward)

Acknowledge All

New Orders Placed on 09/18/23 at 1638 — Acknowledge Section

Ordered	Order	Ordering Provider	
09/18/23 1638	Wound ostomy eval and treat Start: 09/18/23 0730, End: 09/18/23 0730, ONCE, Routine<br>**Last continued at transfer on Sun Sep 17, 2023 5:00 PM**	Whitecoat, Walt, MD	Acknowledge New

New Orders Placed on 09/18/23 at 0703 — Acknowledge Section

Ordered	Order	Ordering Provider	
09/18/23 0703	Glucose monitor nursing POCT- At 0200 IF on insulin. If PO (eating meals or on bolus enteral feedings): Start: 09/18/23 0700, EFFECTIVE NOW, Routine<br>Comments: Do NOT correct this value.	Whitecoat, Walt, MD	Acknowledge New
09/18/23 0703	Carbohydrate Counting by Nursing Start: 09/18/23 0700, EFFECTIVE NOW, Routine	Whitecoat, Walt, MD	Acknowledge New
09/18/23 0703	Patient education - Diabetes Start: 09/18/23 0700, EFFECTIVE NOW, Routine<br>Comments: Basic Survival Skills	Whitecoat, Walt, MD	Acknowledge New
09/18/23 0703	Diabetes Educator IP Consult Start: 09/18/23 0700, End: 09/18/23 0700, ONE TIME, Routine<br>Provider: (Not yet assigned)	Whitecoat, Walt, MD	Acknowledge New

Hyperspace - SJN P2 - PLYUPG cp PREPUPG - SIDNEY-IPRN K.

Epic | Kristoff, Kingsley | Pt Station | My Reports | Resources | Census | Compass Reporting | In Basket | Learning Library | Print | Log Out

Kristoff, Kingsley
Male, 25 year old, 9/16/19...
MRN: 115000002

Bed: TRN IPCD Med...
Cur Location: SH DIA...
Code: Full Code...

None
None
Isolation: None

BestPractice Advisory... | Attending: Whitecoat, Wa... | Allergies: Penicillins, ... | Primary Problem: Sep... | Specimen Collection:... | Admitted: 9/17/2023 (1 d) | Sepsis (H) | Height: 188 cm... | Last Wt: 113.4... | 3 more | Acknowledge Orders (... | New Results, Last... | Active Meds (27) | Social Determinants | Some not on file | Nurses:...

Summary

Nursing Summary | Inpatient Active Orders | Event Log | Intake/Output

Nursing Summary | Off

Select Time Range

Provider: (Not yet assigned)

09/18/23 0703	**Assess for hypoglycemia symptoms** Start: 09/18/23 0700, PER UNIT ROUTINE, Routine<br>Comments: ADRENERGIC Symptoms: Diaphoresis, tremor, tachycardia, hypotension, anxiety, hunger, pallor, increased respirations, circumoral tingling, tingling in extremities, possible nausea/vomiting NEUROGLYCOPENIC Symptoms: Dizziness, headache, clouding of vision, blunted mental activity, loss of fine motor skills, confusion, slurred speech, abnormal behavior, numbness, fatigue/sleepiness, convulsions/seizures, loss of consciousness	Whitecoat, Walt, MD	Acknowledge New
09/18/23 0703	**Glucose monitor nursing POCT** Start: Unscheduled, PRN, Routine<br>Comments: For symptoms of hypoglycemia	Whitecoat, Walt, MD	Acknowledge New
09/18/23 0703	**Glucose monitor nursing POCT** Start: Unscheduled, PRN, Routine<br>Comments: Every 15 Minutes during treatment for hypoglycemia until patient stabilized and glucose value greater than 100 mg/dL	Whitecoat, Walt, MD	Acknowledge New
09/18/23 0703	**glucose gel 15-30 g** Start: 09/18/23 0653, 15-30 g, Oral, EVERY 15 MIN PRN, Routine<br>PRN Reasons: hypoglycemia<br>Admin Instructions: Give first dose for initial blood glucose less than 70 mg/dL per the dosing instructions below. If blood glucose at 15 minute rechecks is still less than or equal to 100 mg/dL, continue to administer doses per blood glucose parameters every 15 minutes, as needed, until blood glucose level is above 100 mg/dL. Dosing Instructions: ~If patient is conscious and able to swallow and NO enteral tube For initial BG 51-69mg/dL OR 15 minute recheck BG 51- 100 mg/dL - give 15 g For BG less than or equal to 50 mg/dL - give 30 g ~ If Enteral tube For initial BG 51-69mg/dL OR 15 minute recheck BG 51- 100 mg/dL - give apple juice 120 mL (4 oz or 15 g of CHO) via enteral tube For BG less than or equal to 50 mg/dL - Give apple juice 240 mL (8 oz or 30 g of CHO) via enteral tube ~Oral gel is preferable for conscious and able to swallow patient. ~IF gel unavailable or patient refuses may provide apple juice per Enteral tube dosing instructions. Document juice on I and O flowsheet.<br>"Or" Linked Group Details	Whitecoat, Walt, MD	Acknowledge New
09/18/23 0703	**dextrose 50 % injection 25-50 mL** Start: 09/18/23 0653, 25-50 mL, Intravenous, EVERY 15 MIN PRN, Routine<br>PRN Reasons: hypoglycemia<br>Admin Instructions: Use if have IV access, BG less than 70 mg/dL and meet dose criteria below: Dose if conscious and alert (or disorientated) and NPO = 25 mL Dose if unconscious / not alert = 50 mL Give first dose for initial blood glucose less than 70 mg/dL. If blood glucose at 15 minute recheck is less than or equal to 100 mg/dL continue to administer carbohydrate treatment every 15 minutes, as needed, based on blood glucose and assessment parameters until blood glucose level is above 100 mg/dL. Vesicant. For ordered doses up to 25 g, give IV Push undiluted. Give each 5g over 1 minute.<br>"Or" Linked Group Details	Whitecoat, Walt, MD	Acknowledge New
09/18/23 0703	**glucagon injection 1 mg** Start: 09/18/23 0653, 1 mg, Subcutaneous, EVERY 15 MIN PRN, Routine<br>PRN Reasons: hypoglycemia (May repeat x 1 only)<br>Admin Instructions: May give SQ or IM. ONLY use glucagon IF patient has NO IV access AND is UNABLE to swallow AND blood glucose is LESS than or EQUAL to 50 mg/dL. If ordered IV, give IV Push over 1 minute. Reconstitute with 1mL sterile water.<br>"Or" Linked Group Details	Whitecoat, Walt, MD	Acknowledge New
09/18/23 0703	**Notify Provider** Start: 09/18/23 1315, EFFECTIVE NOW, Routine<br>Comments: IF severe hypoglycemic episodes (Blood Glucose less than or equal to 50) and review insulin/oral hypoglycemic agents and caloric sources (PO intake, CHO units, IV fluid, TPN, Enteral Feedings).	Whitecoat, Walt, MD	Acknowledge New
09/18/23 0703	**insulin aspart (NovoLOG) injection (RAPID ACTING)** Start: 09/18/23 0730, 1-7 Units, Subcutaneous, 3 TIMES DAILY BEFORE MEALS, Routine<br>Admin Instructions: Correction Scale - MEDIUM INSULIN RESISTANCE DOSING Do Not give Correction Insulin if Pre-Meal BG less than 140. For Pre-Meal BG 140 - 189 give 1 unit. For Pre-Meal BG 190 - 239 give 2 units. For Pre-Meal BG 240 - 289 give 3 units. For Pre-Meal BG 290 - 339 give 4 units. For Pre-Meal BG 340- 399 give 5 units. For Pre-Meal BG 400-449 give 6 units For Pre-Meal BG greater than or equal to 450 give 7 units. To be given with prandial insulin, and based on pre-meal blood glucose. Notify provider if glucose greater than or equal to 350 mg/dL after administration of correction dose. If given at mealtime, administer within 30 minutes of start of meal	Whitecoat, Walt, MD	Acknowledge New
09/18/23 0703	**insulin aspart (NovoLOG) injection (RAPID ACTING)** Start: 09/18/23 2100, 1-5 Units, Subcutaneous, AT BEDTIME, Routine<br>Admin Instructions: MEDIUM INSULIN RESISTANCE DOSING Do Not give Bedtime Correction Insulin if BG less than 200. For BG 200 - 249 give 1 units. For BG 250 - 299 give 2 units. For BG 300 - 349 give 3 units. For BG 350 -399 give 4 units. For BG greater than or equal to 400 give 5 units. Notify provider if glucose greater than or equal to 350 mg/dL after administration of correction dose. If given at mealtime, administer within 30 minutes of start of meal	Whitecoat, Walt, MD	Acknowledge New
09/18/23 0703	**CBC with Platelets & Differential** Start: 09/18/23 0900, End: 09/18/23 0900, ROUTINE, Routine	Whitecoat, Walt, MD	Acknowledge New
09/18/23 0703	**Basic metabolic panel** Start: 09/18/23 0900, End: 09/18/23 0900, ROUTINE, Routine	Whitecoat, Walt, MD	Acknowledge New
09/18/23 0703	**UA with Microscopic reflex to Culture** Start: 09/18/23 0900, End: 09/18/23 0900, ROUTINE, Routine	Whitecoat, Walt, MD	Acknowledge New
09/18/23 0703	**Glucose monitor nursing POCT- IF PO (eating meals or on bolus enteral feedings), within 30 minutes prior to each meal and at bedtime.** Start: 09/18/23 0700, 4 TIMES DAILY BEFORE MEALS & AT BEDTIME, Routine	Whitecoat, Walt, MD	Acknowledge New
09/18/23 0703	**Vital signs** Start: 09/18/23 0800, End: 09/18/23 1400, EVERY 2 HOURS, Routine	Whitecoat, Walt, MD	Acknowledge New
09/18/23 0703	**Peripheral IV catheter** Start: 09/18/23 0700, EFFECTIVE NOW, Routine [illegible]	Whitecoat, Walt, MD	Acknowledge New

70°F
Sunny
Search
ENG
11:37 AM
9/18/2023

Hyperspace - SJN P2 - PLYUPG cp PREPUPG - SIDNEY-IPRN K.

Epic | Kristoff, Kingsley | Pt Station | My Reports | Resources | Census | Compass Reporting | In Basket | Learning Library | Print | Log Out

Kristoff, Kingsley
Male, 25 year old, 9/16/19...
MRN: 115000002

Bed: TRN IPCD Med...
Cur Location: SH DIA...
Code: Full Code...

None
None
Isolation: None

BestPractice Advisory... | Attending: Whitecoat, Wa... | Allergies: Penicillins, ... | Primary Problem: Sep... | Specimen Collection:... | Admitted: 9/17/2023 (1 d) | Sepsis (H) | Height: 188 cm... | Last Wt: 113.4... | 3 more | Acknowledge Orders (... | New Results, Last... | Active Meds (27) | Social Determinants | Some not on file | Nurses:...

Summary

Nursing Summary | Inpatient Active Orders | Event Log | Intake/Output

Nursing Summary

Select Time Range

	Comments: Insert and maintain peripheral IV. Site care and add-on device change per Fairview Vascular Access Device Guidelines. Not routinely used for blood draws.		
09/18/23 0703	lidocaine 1 % 0.1-1 mL Start: 09/18/23 0659, 0.1-1 mL, Other, EVERY 1 HOUR PRN, Routine<br>PRN Reasons: mild pain with VAD insertion<br>Admin Instructions: MAX dose 1 mL subcutaneous OR intradermal along the side of the vein in divided doses as needed for VAD insertion. Do NOT give if patient has a history of allergy to any local anesthetic or any "caine" product. Do NOT use both lidocaine intradermal/subcutaneous injection and the lidocaine cream on the same site.	Whitecoat, Walt, MD	Acknowledge New
09/18/23 0703	lidocaine (LMX4) kit Start: 09/18/23 0659, Topical, EVERY 1 HOUR PRN, Routine<br>PRN Reasons: pain (with VAD insertion)<br>Admin Instructions: Apply at least 30 minutes prior to VAD insertion in divided doses as needed for size of site for insertion. MAX Dose: 2.5 g (½ of 5 g tube) Do NOT give if patient has a history of allergy to any local anesthetic or any "caine" product. Do NOT use both lidocaine intradermal/subcutaneous injection and the lidocaine cream on the same site.	Whitecoat, Walt, MD	Acknowledge New
09/18/23 0703	sodium chloride (PF) 0.9% PF flush 3 mL Start: 09/18/23 0730, 3 mL, Intracatheter, EVERY 8 HOURS, Routine<br>Admin Instructions: to lock peripheral IV dormant line	Whitecoat, Walt, MD	Acknowledge New
09/18/23 0703	sodium chloride (PF) 0.9% PF flush 3 mL Start: 09/18/23 0659, 3 mL, Intracatheter, EVERY 1 MIN PRN, Routine<br>PRN Reasons: line flush, other (to ensure patency or to lock dormant line)	Whitecoat, Walt, MD	Acknowledge New
09/18/23 0703	Lactic acid whole blood Start: 09/18/23 0700, End: 09/18/23 0700, STAT, STAT	Whitecoat, Walt, MD	Acknowledge New
09/18/23 0703	cefTRIAXone (ROCEPHIN) 2 g vial to attach to NS 100 ml bag for ADULTS or NS 50 ml bag for PEDS Start: 09/18/23 0730, 2 g, Intravenous, EVERY 24 HOURS, STAT<br>Admin Instructions: If not given in ED, first dose STAT and to be started on the floor ordered PRIOR to transfer.	Whitecoat, Walt, MD	Acknowledge New
09/18/23 0703	Pharmacy to dose vancomycin Start: 09/18/23 0715, End: 09/18/23 0715, ONE TIME, STAT<br>Comments: First Dose STAT - if not given in ED.<br>Provider: (Not yet assigned)	Whitecoat, Walt, MD	Acknowledge New

New Discontinued Orders

Acknowledge Section

Discontinued		Discontinuing Provider	
09/18/23 0703	Lactic acid - subsequent sepsis screen if first result of paired test is >= 2 mmol/L Start: 09/17/23 0700, End: 09/17/23 0700, STAT, STAT, Status: Canceled<br>Comments: Only necessary if first lactic acid result from paired order is >= 2 mmol/L. May discontinue this order if prior lactate result is < 2 mmol/L.	Foley, Maurice, RN	Acknowledge Discontinue
09/18/23 0703	insulin aspart (NovoLOG) injection (RAPID ACTING) Start: 09/17/23 1800, End: 09/18/23 0703, 0-16 Units, Subcutaneous, 3 TIMES DAILY WITH MEALS, Routine, Discontinue Reason: Error, Status: Discontinued<br>Admin Instructions: For BG < 70, follow hypoglycemia protocol and notify ordering provider. If patient can eat or drink, give oral carbohydrate as ordered per hypoglycemia protocol. If patient NPO, give dextrose 50 % IV as ordered per hypoglycemia protocol. If NPO and no IV access, give glucagon IM as ordered per hypoglycemia protocol. Check BG every 15 minutes and repeat treatment if continued BG < 80. If given at mealtime, administer within 30 minutes of start of meal	Foley, Maurice, RN	Acknowledge Discontinue

Acknowledge All

MY SHIFT (Orders and Tasks)

Additional Orders/Task Report

- ACKNOWLEDGE ORDERS
- ALL SIGNED AND HELD ORDERS
- MEDS TO COSIGN
- PERI-OP/PROCEDURE ORDERS
- INPATIENT ACTIVE ORDERS
- CONDITIONAL ORDERS
- ORDER HISTORY

Blood Transfusion

- TRANSFUSION ORDERS
- TRANSFUSION REPORT (Timeline)
- TRANSFUSION HISTORY

MY PATIENT

Additional Patient Report Links

- ADT
- CARE PLAN OVERVIEW
- ED NOTES
- ONCOLOGY SPRINGBOARD
- CARE PLAN ACCORDION (Timeline)
- ED ENCOUNTER SUMMARY
- IP/ED/OB Preop Checklist
- TREATMENT TEAM

Hospital Problems

Comment

Hospital Problem List — Date Reviewed: 9/16/2023

ICD-10-

CURRENT STATUS

Discharge Planning and Interdisciplinary Care

- Rehab Details (Timeline)
- Care Management
- Discharge Planner

Vitals

Timeline

View Table

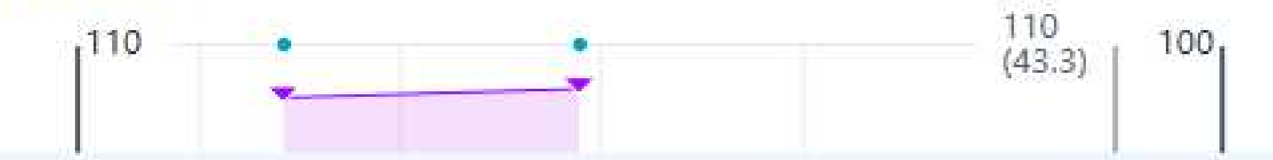

70°F Sunny | Search | ENG | 11:38 AM 9/18/2023

Hyperspace - SJN P2 - PLYUPG cp PREPUPG - SIDNEY-IPRN K.

Epic | Kristoff, Kingsley | Pt Station | My Reports | Resources | Census | Compass Reporting | In Basket | Learning Library | Print | Log Out

Kristoff, Kingsley
Male, 25 year old, 9/16/19...
MRN: 115000002

Bed: TRN IPCD Med...
Cur Location: SH DIA...
Code: Full Code...

None
None
Isolation: None

BestPractice Advisory...
Attending: Whitecoat, Wa...
Allergies: Penicillins, ...
Primary Problem: Sep...
Specimen Collection:...
Admitted: 9/17/2023 (1 d)
Sepsis (H)
Height: 188 cm...
Last Wt: 113.4...
3 more
Acknowledge Orders (...
New Results, Last...
Active Meds (27)
Social Determinants
Some not on file
Nurses:...

Summary

Nursing Summary | Inpatient Active Orders | Event Log | Intake/Output

Nursing Summary

Select Time Range

Blood Transfusion

TRANSFUSION ORDERS | TRANSFUSION REPORT (Timeline) | TRANSFUSION HISTORY

Recent Lab Results for RN Managed Orders

Timeline

(Last result from the past 72 hours)

Switch View

Potassium
5.2
09/17 0730

POCT Orders

Expand | Hide

(From admission, onward)

Start		Ordered
09/18/23 0700	Glucose monitor nursing POCT- At 0200 IF on insulin. If PO (eating meals or on bolus enteral feedings): EFFECTIVE NOW Comments: Do NOT correct this value.	09/18/23 0703
09/18/23 0700	Glucose monitor nursing POCT- IF PO (eating meals or on bolus enteral feedings), within 30 minutes prior to each meal and at bedtime. 4 TIMES DAILY BEFORE MEALS & AT BEDTIME	09/18/23 0703
09/17/23 1700	POCT glucose meter docked device 4 TIMES DAILY BEFORE MEALS & AT BEDTIME Comments: Notify provider if patient bec...	09/17/23 1419
Unscheduled	Glucose monitor nursing POCT PRN Comments: For symptoms of hypoglycemia	09/18/23 0703
Unscheduled	Glucose monitor nursing POCT PRN Comments: Every 15 Minutes during treatm...	09/18/23 0703

Unit Specimen Collection

Ordered
09/18/23 0704

UA with Microscopic reflex to Culture - ROUTINE, Prio: Routine, Needs to be Collected

Scheduled	Task	Status
09/18/23 0900	Print Label for UA with Microscopic reflex to Culture	Incomplete

Order Class: Unit Collect

Click Here to See Labs Due the Next 7 Days

Labs Next 7 days

Hospital Problems

Comment

Hospital Problem List

Date Reviewed: 9/16/2023

	ICD-10-CM	Priority	Class	Noted
Sepsis (H)	A41.9	Medium		9/16/2023
Abscess of bursa of right knee	M71.061	Medium		9/16/2023
Mild intermittent asthma with acute exacerbation (Chronic)	J45.21	Medium		9/16/2023
Uncontrolled diabetes mellitus (H) (Chronic)	E11.65	Medium		9/16/2023

Emergency Contacts

No emergency contacts on file.

Admission Information

None

Getting to Know Me

None

Patient Care Coordination Note

No Care Coordination Notes on File

Social Determinants of Health

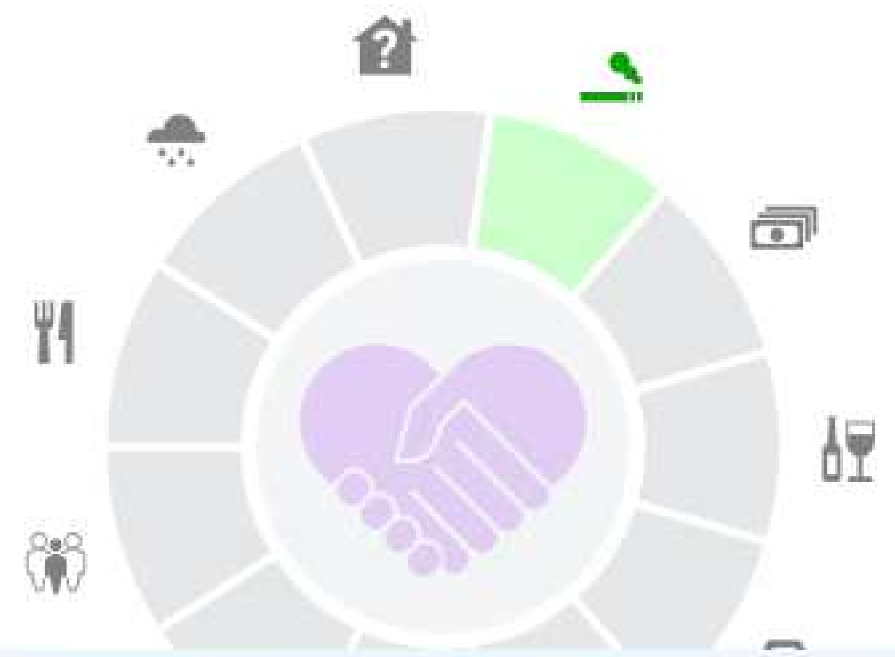

View Table

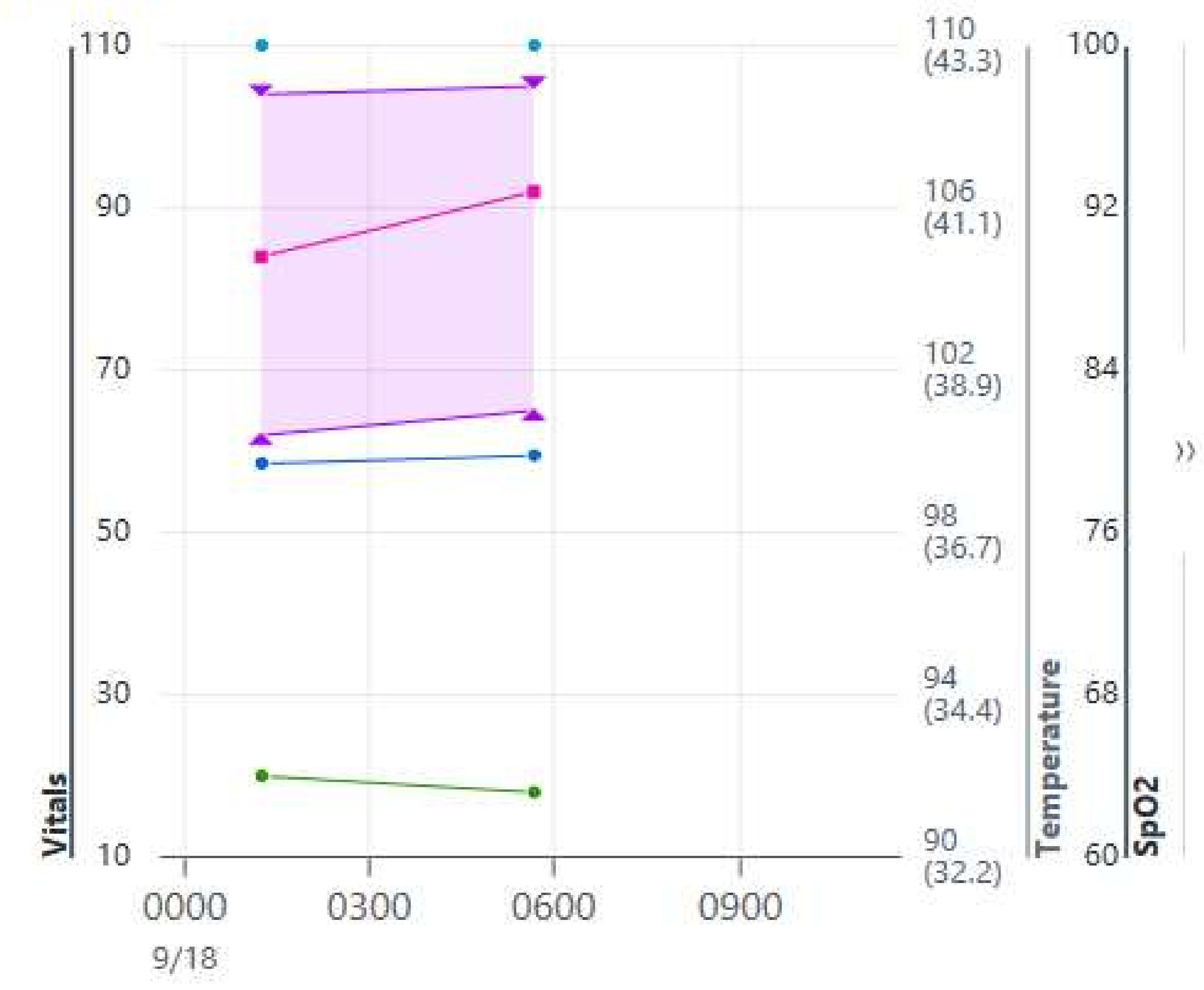

Pain Last 24 hours

Report

None

Pain (Secondary Site)

Report

None

Intake/Output

Report

View Table

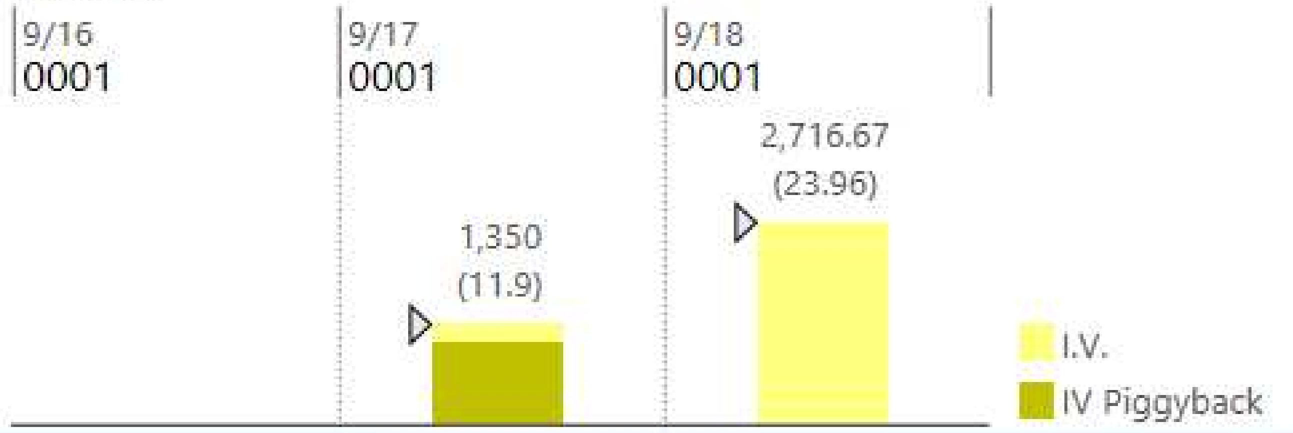

70°F Sunny | Search | ENG | 11:38 AM 9/18/2023

Hyperspace - SJN P2 - PLYUPG cp PREPUPG - SIDNEY-IPRN K.

Epic | Kristoff, Kingsley | Pt Station | My Reports | Resources | Census | Compass Reporting | In Basket | Learning Library | Print | Log Out

Kristoff, Kingsley
Male, 25 year old, 9/16/19...
MRN: 115000002

Bed: TRN IPCD Med...
Cur Location: SH DIA...
Code: Full Code...

None
None
Isolation: None

BestPractice Advisory...
Attending: Whitecoat, Wa...
Allergies: Penicillins, ...
Primary Problem: Sep...
Specimen Collection:...
Admitted: 9/17/2023 (1 d)
Sepsis (H)
Height: 188 cm...
Last Wt: 113.4...
3 more
Acknowledge Orders (...
New Results, Last...
Active Meds (27)
Social Determinants
Some not on file
Nurses:...

Summary

Nursing Summary | Inpatient Active Orders | Event Log | Intake/Output

Nursing Summary | Off

Select Time Range

Click Here to See Labs Due the Next 7 Days

Labs Next 7 days

RT Specimen Collection

None

Diet Order

Collapse | Hide

(9999h ago, onward)

Start: 09/17/23 1420
Adult diet Regular DIET EFFECTIVE NOW
References: The IDDSI Framework
Question: Answer: Regular
Ordered: 09/17/23 1419

Activity Order

Collapse | Hide

(9999h ago, onward)

Start: 09/17/23 1701
Activity (specify) No Restrictions UNTIL DISCONTINUED
References: ADULT ICU Early Mobility Protocol
Question: Answer: No Restrictions
Ordered: 09/17/23 1700

Verify Pump Settings

None

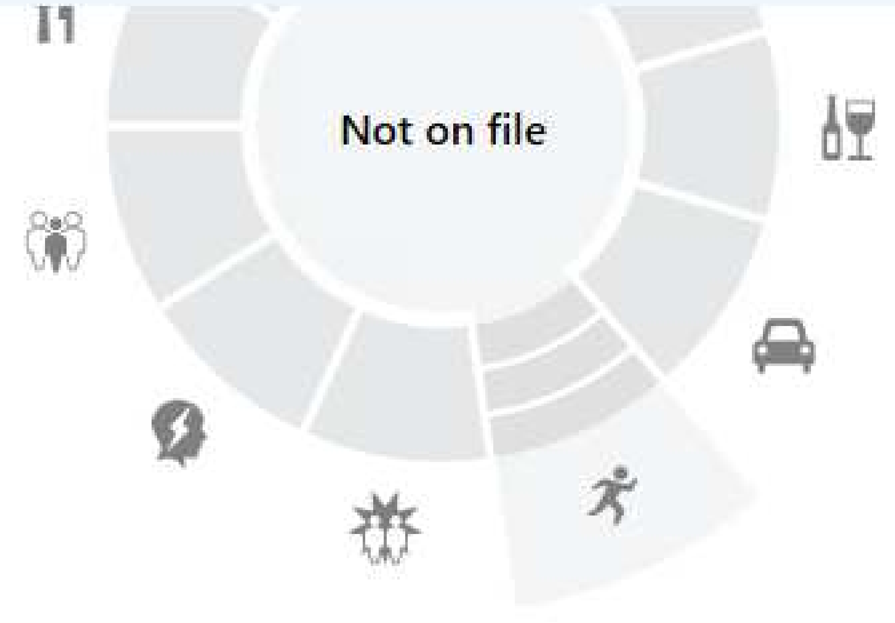

Key Historical Information/Social Determinants

None

Significant Events

None

RRT

None

About Me (Individualization)

Notes

09/17 1421 Care Plan by Foley, Maurice, RN Note

Scales and Screens Documentation

Scales and Screens

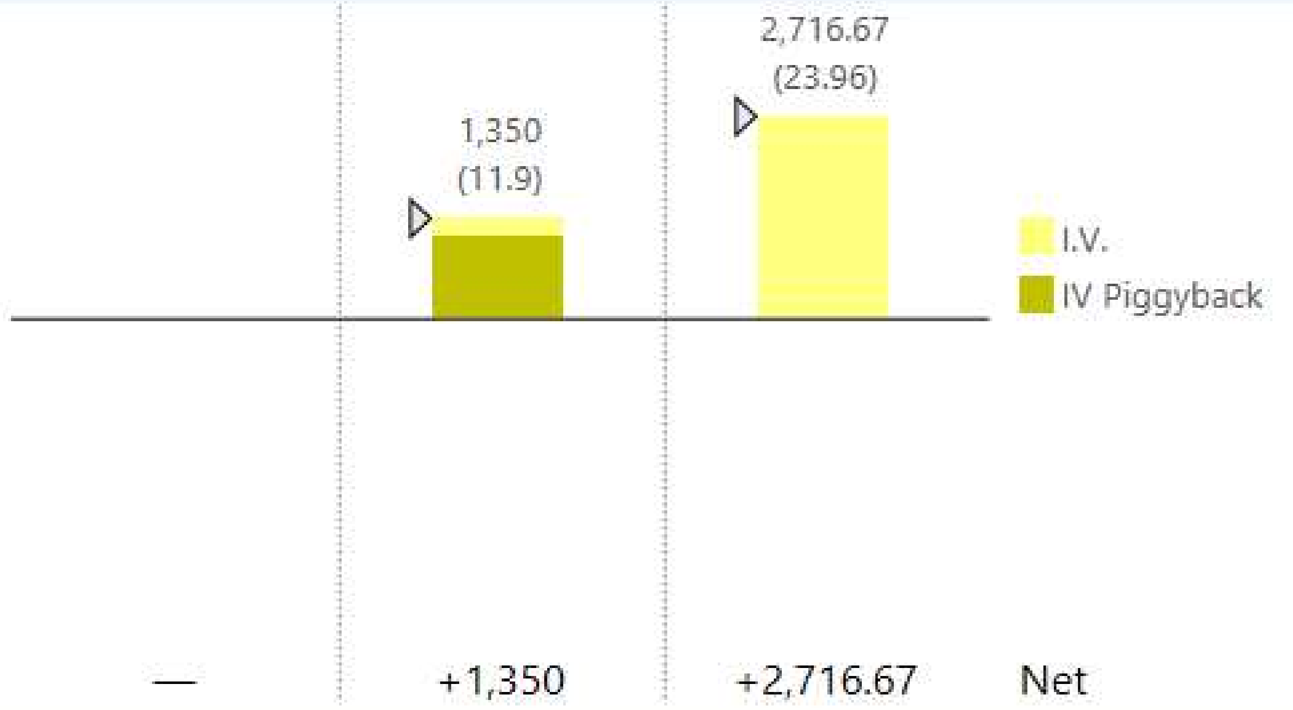

Weight

Timeline

Weight

09/17 0645 113.4 kg (250 lb)

Respiratory

Timeline

Blood Gases (Last 24 hours)

None

Respiratory Data (Last 24 hours)

None

Skin

None

Lines/Drains/Airways

Timeline

Switch View

Lines | Wounds

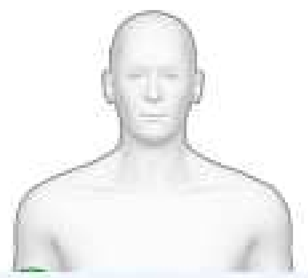

Peripheral IV 9/17/2023 Anterior;Right Upper... 1 day

Peripheral IV 9/17/2023 Anterior;Left Other (C... 1 day

Wound Knee Abscess 1 day

70°F
Sunny
Search
ENG
11:39 AM
9/18/2023

Hyperspace - SJN P2 - PLYUPG cp PREPUPG - SIDNEY-IPRN K.

Epic | Kristoff, Kingsley | Pt Station | My Reports | Resources | Census | Compass Reporting | In Basket | Learning Library | Print | Log Out

Kristoff, Kingsley
Male, 25 year old, 9/16/19...
MRN: 115000002

Bed: TRN IPCD Med...
Cur Location: SH DIA...
Code: Full Code...

None
None
Isolation: None

BestPractice Advisory...
Attending: Whitecoat, Wa...
Allergies: Penicillins, ...
Primary Problem: Sep...
Specimen Collection:...
Admitted: 9/17/2023 (1 d)
Sepsis (H)
Height: 188 cm...
Last Wt: 113.4...
3 more
Acknowledge Orders (...
New Results, Last...
Active Meds (27)
Social Determinants
Some not on file
Nurses:...

Summary

Nursing Summary | Inpatient Active Orders | Event Log | Intake/Output

Nursing Summary | Off

Select Time Range

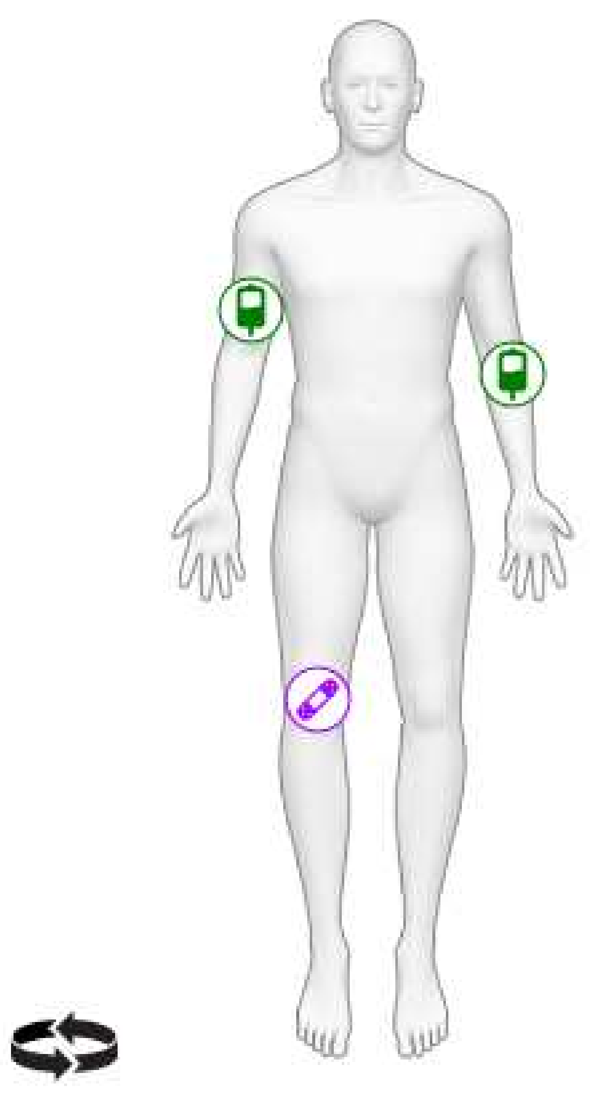

Peripheral IV 9/17/2023 Anterior;Right Upper...		1 day
Peripheral IV 9/17/2023 Anterior;Left Other (C...		1 day
Wound Knee Abscess		1 day

LAB / RESULTS

Lab Reports

2 HOUR OVERVIEW (Timeline)
ELECTROLYTE REPLACEMENT (Timeline)
LABS SINCE ADMISSION (Timeline)
LABS SINCE MIDNIGHT (Timeline)
MICROBIOLOGY
REPRINT LAB LABEL
UNRESULTED LABS

Differential and INR

Timeline

(Last result from the past 72 hours)

Switch View

None

Renal (I/O Link)

Timeline

BMP Results (Up to last 3 results from past 72 hours)

	09/17 0730
Sodium	139
Potassium	5.2
Chloride	100
Carbon Dioxide	15
Urea Nitrogen	10

Cardiac

Timeline

(Last result from the past 72 hours)

Switch View

None

Hgb, WBC, Platelets

Timeline

(Last result from the past 72 hours)

Switch View

None

Liver Function

Timeline

(Last result from the past 72 hours)

Switch View

None

EKG Results

EKG Results

70°F
Sunny

Search

ENG

11:39 AM
9/18/2023

Hyperspace - SJN P2 - PLYUPG cp PREPUPG - SIDNEY-IPRN K.

Epic | Kristoff, Kingsley | Pt Station | My Reports | Resources | Census | Compass Reporting | In Basket | Learning Library | Print | Log Out

Kristoff, Kingsley
Male, 25 year old, 9/16/19...
MRN: 115000002

Bed: TRN IPCD Med...
Cur Location: SH DIA...
Code: Full Code...

None
None
Isolation: None

BestPractice Advisory...
Attending: Whitecoat, Wa...
Allergies: Penicillins, ...
Primary Problem: Sep...
Specimen Collection:...
Admitted: 9/17/2023 (1 d)
Sepsis (H)
Height: 188 cm...
Last Wt: 113.4...
3 more
Acknowledge Orders (...
New Results, Last...
Active Meds (27)
Social Determinants
Some not on file
Nurses:...

Summary

Nursing Summary | Inpatient Active Orders | Event Log | Intake/Output

Nursing Summary | Off

Select Time Range

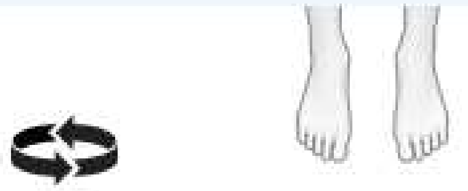

LAB / RESULTS

Lab Reports

2 HOUR OVERVIEW (Timeline)
ELECTROLYTE REPLACEMENT (Timeline)
LABS SINCE ADMISSION (Timeline)
LABS SINCE MIDNIGHT (Timeline)
MICROBIOLOGY
REPRINT LAB LABEL
UNRESULTED LABS

Differential and INR

Timeline

(Last result from the past 72 hours)

Switch View

None

Renal (I/O Link)

Timeline

BMP Results (Up to last 3 results from past 72 hours)

	09/17 0730
Sodium	139
Potassium	5.2
Chloride	100
Carbon Dioxide	15
Urea Nitrogen	10

Cardiac

Timeline

(Last result from the past 72 hours)

Switch View

None

Hgb, WBC, Platelets

Timeline

(Last result from the past 72 hours)

Switch View

None

Liver Function

Timeline

(Last result from the past 72 hours)

Switch View

None

EKG Results

EKG Results

Radiology

Report

(Last 720 hours)

None

SURGICAL / PROCEDURAL / FUTURE APPTS

Surgical/Procedural Cases on this Admission

No scheduled surgical procedures for this admission

Appointments for Next 3 Days

Comment

9/18/2023 - 9/21/2023

None

70°F
Sunny
Search
ENG
11:39 AM
9/18/2023
