# Supplementary material for: Exploratory Co-Design on Electronic Health Record Nursing Summaries: Case Study
Source: JMIR Form Res. 2025 Mar 11;9:e68906. doi: 10.2196/68906 (PMC11918977; doi:10.2196/68906)
Supplement: Multimedia Appendix 2 [file formative-v9-e68906-s002.docx]

**Supplementary File 2.** Information Types in Nursing Summaries

| Heading of information types | Contents |
| --- | --- |
| About me | Individualized notes that nurses use to track patient care (e.g., foley care) |
| Activity order | Order regarding the level of physical activity a patient can engage in |
| Additional links | Navigational links for CRRT, ECMO, cardiac cath. history, dialysis, code blue, etc. |
| Admission information | Notes regarding the patient’s admission history |
| Appointments | Scheduled appointments for next 3 days |
| Blood transfusion | Navigational links for blood transfusion orders, reports, and history |
| Cardiac results | Cardiac exam results |
| Care coordination note | Notes on collaboration and communication between clinicians |
| Discharge plans | Navigational links for discharge plans and interdisciplinary care management |
| Diet order | Order regarding the type of food and nutritional restrictions a patient should follow |
| Differential/INR | Laboratory exam results for the differential and INR from the past 72 hours |
| EKG results | Navigational link for EKG results |
| Emergency contacts | Contact information in case of an emergency |
| Getting to know me | Notes on the patient’s care preference (e.g., preferred intravenous injection site) |
| Hgb, WBC, platelets | Laboratory exam results for Hgb, WBC, platelets |
| Hospital problems | List of medical diagnoses, ICD-10-CM codes, and priority |
| Intake/output | The amount and the trend of intake and output |
| Key history/social determinants | Notes on patient’s medical history, lifestyle, and socioeconomic status |
| Labs due in the next 7 days | Scheduled laboratory tests that need to be performed within the upcoming week |
| Lines/drains/airways | An avatar displaying the location, types, and descriptions of lines, drains, and airways |
| Liver function | Liver function test results |
| Lab reports | Navigational links for lab trends, microbiology, unresulted labs, lab label reprint, etc. |
| Orders | Active orders to be acknowledged |
| Orders/task links | Navigational links for all signed and held orders, meds to cosign, order history, conditional orders, etc. |
| Pain for the last 24 hours | Notes on patient’s perceived pain during the previous day |
| Pain secondary site | Notes on patient’s perceived secondary pain |
| Patient links | Navigational links for treatment team, ED notes, care plans, etc. |
| Point-of-care testing orders | Tests that are conducted at the patient’s bedside such as blood sugar testing |
| Pump settings | Programmed parameters on an intravenous pump |
| Radiology | Radiology exam results |
| Rapid response team | Healthcare professional team who are called to assist in emergency |
| Recent lab results (for nurse-managed orders) | Recent laboratory exam results directly relevant to orders managed by nurses (i.e., electrolyte replacement) |
| Renal results | Laboratory exam results on renal functions |
| Respiratory results | Blood gas results and respiratory data |
| Respiratory specimen | Collection of respiratory swab specimens |
| Scale and screen documentation | Results of standardized assessments or screenings, such as mental health |
| Significant events | Notes about significant events during the admission |
| Skin | Nursing notes regarding the patient’s skin condition |
| Social determinants of health | Economic, social, and environmental information of the patient |
| Sticky notes to physicians | Message to doctors in charge |
| Sticky notes to treatment team | Message to the treatment team |
| Surgical procedures | Scheduled surgical procedures for this admission |
| Unit specimen | Results of the analysis of body fluid, tissue, or urine |
| Vital signs | Value and the trends of pulse, respiration, blood pressure, temperature, oxygen saturation |
| Weight | Daily weight |

*Note.* CRRT: continuous renal replacement therapy; ECMO: extracorporeal membrane oxygenation; INR: international normalized ratio; EKG: electrocardiogram; Hgb: hemoglobin; ICD-10-CM codes: International Classification of Diseases, Tenth Revision, Clinical Modification; WBC: white blood cell.
